# Paired biopsy-surface and tissue microbiome profiling reveals tissue-associated bacterial signatures in nasopharyngeal carcinoma

**DOI:** 10.3389/fcimb.2026.1892233

**Published:** 2026-10-14

**Authors:** Nan Shi, Zongjian Huang, Xuesong Wang, Ying Xie, Anzhou Tang, Wei Xia

**Affiliations:** 1 Department of Otorhinolaryngology Head and Neck Surgery, The First Affiliated Hospital of Guangxi Medical University, Nanning, China; 2 Department of Head and Neck Tumor Surgery, Guangxi Medical University Cancer Hospital, Nanning, China; 3 Key Laboratory of Early Prevention and Treatment for Regional High-Frequency Tumours (Guangxi Medical University), Ministry of Education, Nanning, China; 4 Department of Otorhinolaryngology Head and Neck Surgery, Binzhou Central Hospital, Binzhou, Shandong, China; 5 School of Life Sciences Institute, Guangxi Medical University, Nanning, China; 6 School of International Education, Guangxi Medical University, Nanning, China

**Keywords:** Epstein-Barr virus, immune microenvironment, intratumoral bacteria, microbe and host, nasopharyngeal carcinoma

## Abstract

**Introduction:**

Tumor-associated microbiota have been implicated in cancer biology, but the relationship between bacteria detected on the biopsy surface and those detected within tumor tissue remains poorly defined.

**Methods:**

In this study, we profiled paired biopsy-surface swabs and tissue biopsies from patients with nasopharyngeal carcinoma (NPC) and benign nasopharyngeal lymphoid hyperplasia (NLH) controls using five-region 16S rRNA gene sequencing. Matched bulk RNA-seq data were analyzed to explore associations between microbial features, Epstein-Barr virus (EBV) transcription, host pathway activity, and inferred immune-cell composition.

**Results:**

Tissue-associated microbial profiles showed stronger separation between NPC and NLH than biopsy-surface profiles in this discovery cohort. Several NPC-enriched species-level taxonomic assignments were associated with higher EBV transcript levels, proliferative and cell-cycle pathway activity, and lower inferred CD8 T-cell and plasmacytoid dendritic-cell signatures.

**Discussion:**

These findings indicate that NPC tissue contains microbial signatures that differ from biopsy-surface communities and support further validation of tissue-associated microbe-EBV-host relationships.

## Introduction

The human microbiota has become an important focus in cancer biology because microbial communities can influence inflammation, epithelial signaling, metabolism, and antitumor immunity (Bhatt et al., 2017; Sepich-Poore et al., 2021). These effects are especially relevant at mucosal sites, where host tissues are continuously exposed to commensal and environmental microorganisms. In tumors, bacteria may be detected as surface-associated organisms, tissue-associated communities, or, in selected settings, intracellular populations. Distinguishing these possibilities is essential before assigning biological or clinical meaning to a microbial signal.

Nasopharyngeal carcinoma (NPC) arises in the upper airway mucosa and is strongly linked to Epstein-Barr virus (EBV) (Tsao et al., 2017; El Tekle and Garrett, 2023). The nasopharynx harbors diverse microbial communities, and altered oral or nasopharyngeal microbiota have been reported in patients with NPC (Liao et al., 2024). These observations raise the possibility that microbes, EBV, and local immune conditions interact within the nasopharyngeal niche. However, microbial signals obtained from surface sampling may not reflect the organisms detected in tumor tissue, particularly when the tumor compartment is a low-biomass environment that is vulnerable to contamination and sampling artifacts.

Intratumoral microbiota have been described across several cancer types and may affect cancer-cell behavior, immune responses, and therapy response (El Tekle and Garrett, 2023; Yang et al., 2023; Sepich-Poore et al., 2024). In NPC, prior studies have reported bacterial DNA or bacterial taxa in tumor tissues and have linked intratumoral bacterial burden with clinical outcomes (Qiao et al., 2022; Guo et al., 2024). These studies support the relevance of tumor-associated microbial signals in NPC, but they do not fully resolve whether tissue-detected bacteria are compositionally distinct from organisms present on the lesion surface.

A key unresolved question is therefore how biopsy-surface and tissue-associated microbial communities relate within the same nasopharyngeal lesion. Most studies have profiled either surface samples or tissue samples, rather than paired compartments from the same patient. A paired design can reduce interindividual variability and help determine whether the tumor tissue compartment contains microbial features that are not captured by surface sampling alone.

In this study, we compared microbial profiles from tissue biopsies and matched biopsy-surface swabs from patients with NPC and NLH controls. We used five-region 16S rRNA gene sequencing to characterize bacterial taxonomic profiles and integrated these data with matched bulk RNA-seq to examine exploratory associations with EBV transcripts, host pathway activity, and inferred immune-cell composition. The analysis was designed to identify compartment-specific microbial patterns and to assess whether tissue-associated signatures separate NPC from benign inflammatory controls more clearly than biopsy-surface signatures.

## Materials and methods

### Clinical cohorts and specimen collection

The sequencing cohort comprised 38 newly diagnosed NPC patients and 26 NLH participants. A separate cohort of 24 NPC and 18 NLH participants was used for the initial qPCR assessment of tissue bacterial load. Material from these specimens was exhausted during the initial experiments and was unavailable for subsequent sequencing. The sample size was determined by the availability of eligible participants with paired specimens; no formal *a priori* power calculation was performed. Biopsies were obtained endoscopically using sterile biopsy forceps. After excision, each specimen was gently rinsed with phosphate-buffered saline (PBS) to remove surface debris. The mucosal surface was then sampled using a sterile nylon-flocked swab to obtain a matched biopsy-surface sample. After swabbing, the tissue was rinsed three additional times with PBS. A sterile swab was exposed to ambient air in the operating room for 30 minutes and served as an environmental negative control. All tissue and swab samples were flash-frozen and stored at −80 °C. The inclusion criteria for all participants were: (1) pathologically confirmed NPC (or nasopharyngeal inflammation), (2) age ≥ 18 years and ≤ 80 years, and (3) no prior treatment. Written informed consent was obtained from all the participants. This study was conducted in accordance with the Declaration of Helsinki and was approved by the Ethics Committee of the First Affiliated Hospital of Guangxi Medical University (Approval No. 2022-KY-E273).

### Fluorescence *in situ* hybridization

FISH was performed with Fluorescent *In situ* Hybridization Kits (BOSTER, China). Briefly, the dewaxed and hydrated paraffin sections were incubated in pepsin working solution at 37 °C for 20 min. After treatment in pre-hybridization buffer, sections were incubated in hybridization buffer for 16 h with a pan-bacterial 16S rRNA fluorescent probe (EUB338, Cy3-labeled, sequence 5′-GCTGCCTCCCGTAGGAGT-3′) in a humid and dark environment. The nuclei were counterstained with 4′,6-diamidino-2-phenylindole (DAPI). Sections were imaged using a fluorescence microscope.

### Bacterial culture

Culture experiments were performed using three independent NPC biopsy specimens selected primarily on the basis of biopsy size, as sufficient tissue and cell numbers were required for testing under multiple culture and antibiotic-treatment conditions. Equal volumes of freshly-collected tissue samples were rinsed with sterile phosphate-buffered saline (PBS) to remove surface contaminants. Subsequently, an equivalent volume of PBS was added, and the tissues were homogenized using a tissue grinder to prepare uniform homogenates. Single-cell suspensions were prepared using enzymatic dissociation in RPMI-1640 medium (Gibco, USA) containing 0.5 mg/mL collagenase IV (Gibco, USA) and 1 mg/mL DNase I (Sigma, USA). The final PBS wash following enzymatic dissociation was retained for culture to assess the presence of non-cell-associated bacteria in the wash fluid. This control did not establish the removal of extracellular bacteria adherent to host cells or demonstrate intracellular localization. Aliquots of single-cell suspensions from each donor (n = 3) were incubated overnight under three conditions: without antibiotics, with 200 µg/mL each of ampicillin and gentamicin, or with 10 µg/mL doxycycline. All samples (environmental controls, sterile PBS solution, tissue homogenates, the retained PBS wash, antibiotic-treated suspensions, and untreated suspensions) and were plated on Columbia blood agar (CBA, OXOID, UK) supplemented with 5% sheep blood (Solarbio, China) and incubated aerobically at 37 °C.

### 5R 16S rRNA gene sequencing and bacterial load quantitative polymerase chain reaction

Microbial DNA was extracted from tissue biopsies and paired swabs using a commercial kit (DNeasy PowerSoil Kit; Qiagen, Hilden, Germany), according to the manufacturer’s instructions. A five-region (5R) 16S rRNA gene amplification strategy was used to improve taxonomic resolution in low-biomass samples, and multiplex PCR simultaneously targeted the V2, V3, V5, V6, and V8 hypervariable regions (Davis et al., 2018). Amplicons were purified, quantified using a bioanalyzer, and pooled equimolarly; libraries were prepared using the Illumina 16S Metagenomic Sequencing Kit. Paired-end sequencing (2 × 150 bp) was performed using a NovaSeq 6000. The reads were adapter-trimmed, quality-filtered, de-chimerized, and host (human) reads were removed. Data were processed using QIIME 2 and the SMURF pipeline with the Greengenes reference to generate amplicon sequence variants (ASVs) (Nejman et al., 2020).

Read counts for each sample were normalized, and samples with <1000 total reads (including negative controls) or taxa with relative abundance <10^-4^ were discarded to minimize low-abundance noise. Potential contaminants were assessed using a negative-control-based filtering strategy. Multiple types of negative controls were included, comprising DNA-extraction/PCR negative controls and air-exposed environmental controls collected in the operating room during specimen handling. Contaminant taxa were subsequently identified by their prevalence in negative-control air-sampling libraries: taxa present in ≥50% of controls were deemed contaminants originating from sampling or laboratory workflows. These contaminants were removed from all experimental samples, and the remaining ASV table was considered to represent the true tissue-resident microbiota and used for downstream alpha/beta diversity calculations and differential abundance analyses.

Bacterial load was quantified by qPCR of the tissue DNA extracts using V9 region of universal 16S rRNA primers (Forward 5’-CGGTGAATACGTTCYCGG-3’, Reverse 5’-GGWTACCTTGTTACGACTT-3’, and Probe 5’-CTTGTACACACCGCCCGTC-3’), and copy numbers were estimated from a standard curve (PCR amplification efficiency (E) of 95–105% and correlation coefficient (R^2^) >0.995) generated using serial dilutions of *Escherichia coli* DNA. Takara Premix Ex Taq kit (Takara, Tokyo, Japan) and Bio-Rad CFX-96 instrument (Bio-Rad) were used for qPCR analysis.

### Transcriptomics

Total RNA was extracted using TRIzol (Thermo Fisher Scientific, USA), and its quality was checked using a bioanalyzer. Sequencing libraries (poly(A) selection) were prepared (Illumina TruSeq) and sequenced using an Illumina NovaSeq 6000 (paired-end 150 bp) by the Novogene Bioinformatics Institute (Novogene, China). The reads were quality-trimmed and aligned to the human (hg38) and EBV genomes (NCBI RefSeq accession: GCF_002402265.1, https://www.ncbi.nlm.nih.gov/assembly/GCF_002402265.1) using STAR or HISAT2. Gene counts were obtained using featureCounts and differential expression analysis was performed using DESeq2.

### Cell-type deconvolution

Cellular composition in NPC and NLH tissue samples was estimated from bulk RNA-seq profiles using CIBERSORTx (Newman et al., 2019). The deconvolution reference was based on the annotated single-cell expression profiles from the NPC study by Chen et al. (2020; GEO accession: GSE150430) (Chen et al., 2020). Reference-derived cell-type expression signatures were used to estimate the relative abundance of the represented populations in each tissue sample. Cell-type nomenclature was based on the reference annotations, with CD8^+^ T-cell and dysfunctional CD8^+^ T-cell populations represented separately. The resulting sample-level estimates were compared between NPC and NLH tissues and used in downstream analyses of associations with microbial features. These estimates were interpreted as relative abundances of reference-defined populations rather than direct measurements of cellular differentiation.

### Statistical analysis

Two-group comparisons were performed using two-tailed Student’s *t*-tests for normally distributed data and Wilcoxon rank–sum tests for non-parametric data. Paired data from matched samples (e.g., paired biopsy and surface swabs) were analyzed using paired *t*-tests or Wilcoxon signed–rank tests as appropriate. Multiple-group comparisons were conducted using one-way analysis of variance (ANOVA) or Kruskal–Wallis tests with appropriate *post hoc* corrections. Alpha diversity indices (Chao1 and Shannon) were compared using Wilcoxon rank–sum tests and beta diversity (Bray–Curtis distances) was evaluated using principal coordinate analysis (PCoA) and permutational multivariate analysis of variance (PERMANOVA). Additional PERMANOVA and Shannon diversity analyses included age and sex as covariates and were conducted separately for tissue and biopsy-surface swab samples. These were whole-cohort comparisons, not age- or sex-stratified subgroup analyses. Differential bacterial taxa were identified using Wilcoxon rank–sum tests and confirmed via linear discriminant analysis (LDA) Effect Size (LEfSe). A microbial risk prediction model was constructed using the randomForest package in R and its performance was evaluated using receiver operating characteristic (ROC) curves. All tests were two-sided, and statistical significance was set at *p* < 0.05. Analyses were performed using R software (version 4.2.2).

## Results

### NPC tissues show bacterial signals and culture positivity

To assess whether bacterial signals could be detected in NPC tissue, we combined *in situ*, quantitative, and culture-based approaches. 16S rRNA FISH showed discrete punctate signals in NPC biopsies, whereas such signals were rare in NLH tissues (**Figure 1A**). Inverse-contrast rendering highlighted focal signal accumulation within tissue sections. In the independent bacterial-load cohort, qPCR of tissue DNA extracts detected a median of 9.07 x 10^2^ bacterial 16S copies per ng DNA in NLH (n = 18) and 7.76 x 10^3^ copies per ng DNA in NPC (n = 24; p <0.01; **Figure 1B**). Both tissue groups showed higher signal than the non-template control (NTC) and environmental background control (EBC) (p <0.01), supporting a tissue-associated bacterial signal above measured background. Viability was then examined using tissue homogenates and enzymatically dissociated single-cell suspensions (**Figure 1C**). Cultures yielded bacterial growth from NPC tissue and dissociated cell suspensions, whereas PBS and environmental controls remained sterile (**Figure 1D**; **Supplementary Figure S1**). Growth persisted after exposure to ampicillin plus gentamicin and was suppressed by doxycycline (**Figure 1D**; **Supplementary Figure S1**). These findings support the presence of viable bacteria in NPC tissue and are compatible with a protected or intracellular bacterial fraction.

**Figure 1:**
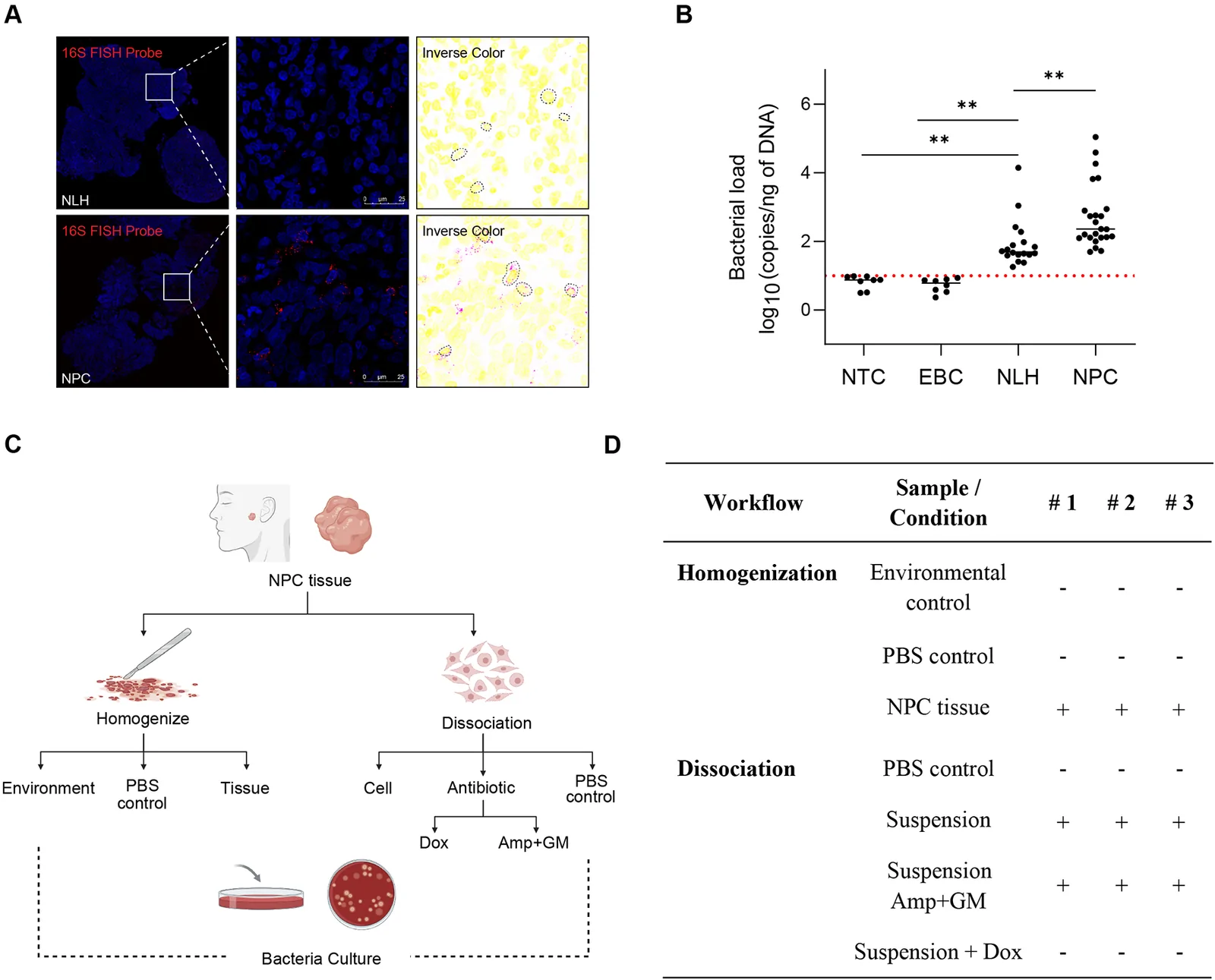
NPC tissues show bacterial signal and culture positivity. **(A)** 16S rRNA fluorescence *in situ* hybridization (FISH) analysis of NPC and benign nasopharyngeal lymphoid hyperplasia (NLH) tissues. NPC tissue sections show more frequent bacterial FISH signal (red) than NLH control tissue. An inverted-color rendering of the FISH channel is provided to visualize focal signal distribution. **(B)** Bacterial load quantified by 16S rDNA qPCR (log10 16S copies per ng DNA). NPC tissue samples showed higher bacterial load than NLH, non-template control (NTC), and environmental background control (EBC). Samples were from a cohort independent of the sequencing cohort (NPC, n = 24; NLH, n = 18). Dots represent individual measurements, and horizontal bars indicate group medians, with statistical significance indicated: ns, not significant; *p <0.05; **p <0.01. **(C)** Viability testing workflow. NPC tissue was processed by homogenization or enzymatic dissociation to single-cell suspensions, followed by culture under the indicated control and antibiotic conditions: cell-impermeable ampicillin plus gentamicin (Amp+GM) or cell-penetrating doxycycline (Dox). Created in BioRender.com. **(D)** Qualitative culture outcomes from three independent NPC biopsy specimens (#1–#3). “Growth detected” (+) and “No growth detected” (-) indicate the presence or absence of visible colony growth under the tested culture conditions. Corresponding plate photographs are shown in **Supplementary Figure S1**.

### NPC tissue-associated microbiota are distinct from NLH tissue and paired biopsy-surface swabs

We next compared the composition of tissue-associated and biopsy-surface microbial profiles in NPC and NLH samples using 16S rRNA sequencing. The 5R 16S strategy was selected because low-biomass samples require broader taxonomic coverage than conventional single-region amplicons (Davis et al., 2018; Nejman et al., 2020). Controls were incorporated during sampling and sequencing workflows to reduce the influence of potential contaminants. Each biopsy was paired with a swab taken from the surface of the freshly excised specimen, enabling comparison between biopsy-surface and tissue-associated profiles within the same lesion (**Figure 2A**; **Supplementary Table S1**). The study included 38 participants with NPC and 26 with benign NLH. Age (mean ± standard deviation) was 44.7 ± 11.7 years in the NPC group and 42.6 ± 14.2 years in the NLH group (P = 0.537). Male participants accounted for 65.8% and 61.5% of the NPC and NLH groups, respectively (P = 0.794). Additional cohort characteristics are provided in **Supplementary Table S2**. Swabs showed higher Chao1 richness and Shannon diversity than paired tissue samples in both groups (p <0.05), consistent with the greater environmental exposure of the mucosal surface. However, the alpha diversity did not differ significantly between NPC and NLH biopsy-surface swabs (**Figure 2B**). In contrast, NPC tissue samples showed higher alpha diversity than NLH tissue samples, as assessed by both the Chao1 and Shannon indices (**Figures 2B, C**; **Supplementary Table S3**). These findings suggest that between-group microbial differences were more evident in tissue-associated profiles than in biopsy-surface swabs within this cohort.

**Figure 2:**
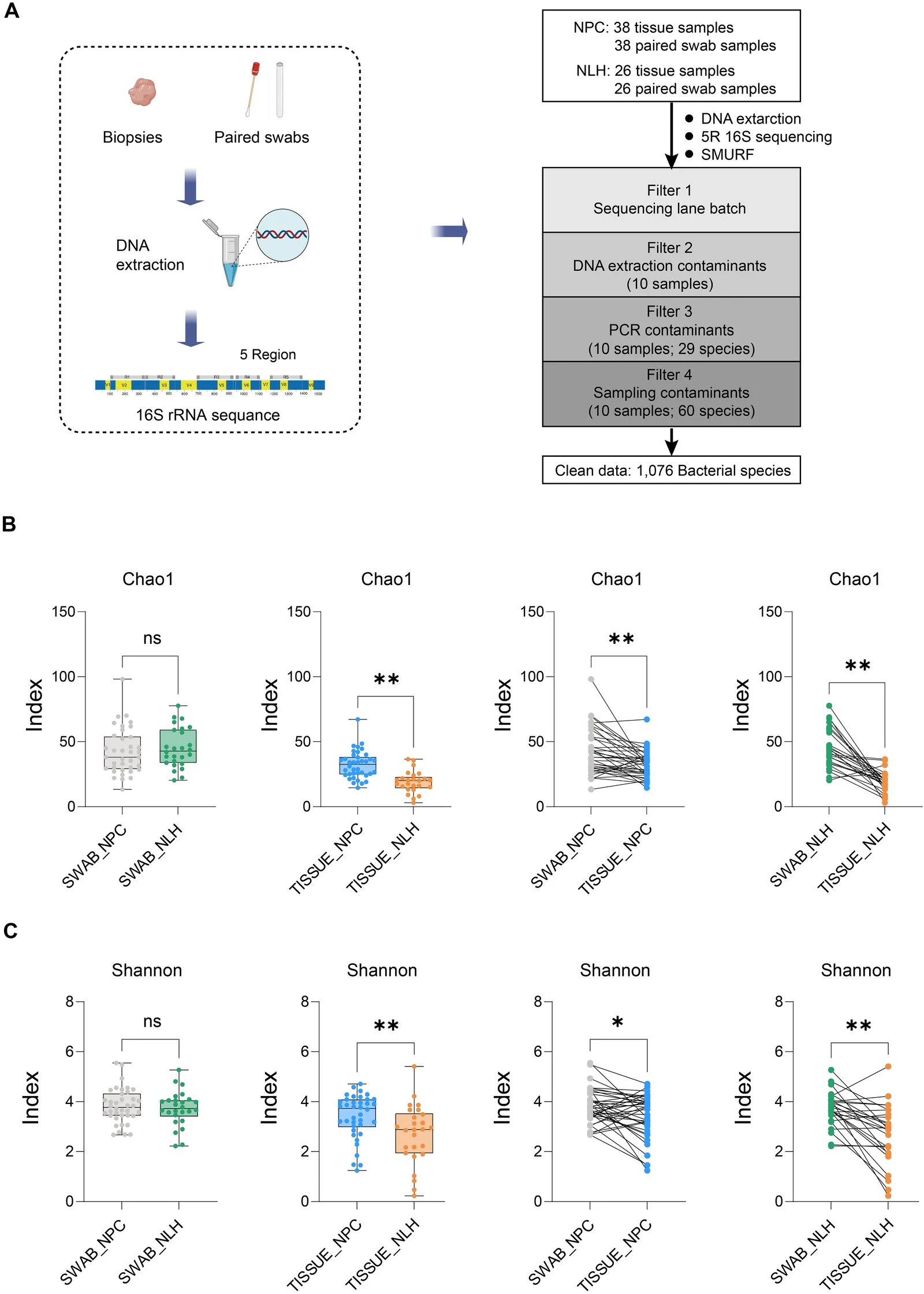
Study design and alpha-diversity contrasts between biopsy-surface and tissue-associated microbiota in NPC. **(A)** Workflow. Biopsies and matched biopsy-surface swabs from NPC (38 tissues, 38 swabs) and NLH controls (26 tissues, 26 swabs) underwent five-region (5R) 16S rRNA sequencing with SMURF-based profiling. A control-filtering workflow was applied to reduce potential lane/batch, DNA-extraction, PCR, and sampling contaminants, yielding 1,076 species-level taxonomic assignments for downstream analysis. Created in BioRender.com. **(B)** Chao1 richness in NPC and NLH tissues and swabs. **(C)** Shannon diversity for the same groups. In the box-and-whisker plots in panels B and C, boxes indicate the interquartile range (25th–75th percentiles), and horizontal lines within boxes indicate the median. Whiskers indicate the most extreme observations within 1.5 times the interquartile range below the first quartile and above the third quartile. Dots represent individual samples; connecting lines indicate paired tissue and biopsy-surface swab samples from the same participant. Statistical significance is indicated as follows: ns, not significant; *p <0.05; **p <0.01. NPC, nasopharyngeal carcinoma; NLH, nasopharyngeal lymphoid hyperplasia; PCR, polymerase chain reaction.

Bray-Curtis PCoA showed broad overlap between NPC and NLH biopsy-surface swab communities (ANOSIM R = -0.038, p = 0.88; **Figure 3A**). In contrast, NPC tissue-associated communities separated from NLH tissue communities, and tissue profiles diverged from paired biopsy-surface profiles within each group (ANOSIM R >0.5, p <0.01; **Figure 3A**). After adjustment for age and sex, disease status remained associated with tissue community composition (Bray-Curtis PERMANOVA pseudo-F = 2.130, R^2^ = 0.033, p <0.001) but not with biopsy-surface swab composition (pseudo-F = 0.994, R^2^ = 0.016, p = 0.391). Multivariate dispersion did not differ by disease group in either compartment. NPC tissue also retained higher Shannon diversity after covariate adjustment (beta = 0.496, 95% CI 0.130-0.862, p = 0.0087), whereas the adjusted swab comparison remained nonsignificant. Thus, within the sampling design used here, microbial differences associated with NPC were more pronounced in tissue specimens than on the surface of the excised biopsy.

**Figure 3:**
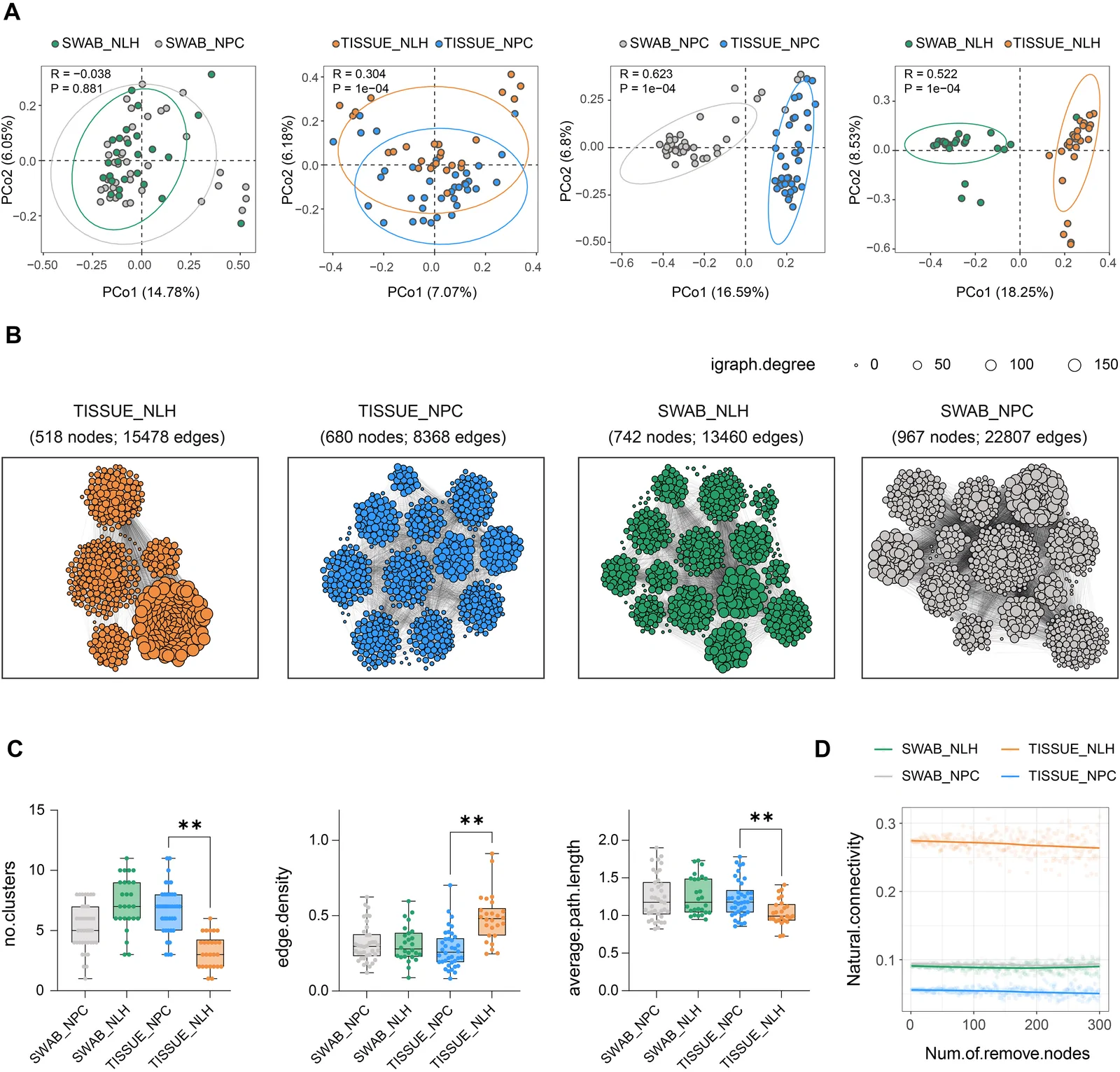
Tissue-associated microbiota diverge from biopsy-surface swabs and show altered inferred network structure in NPC. **(A)** Principal coordinate analysis (PCoA) of Bray-Curtis distances comparing microbiota composition. **(B)** Microbial co-occurrence network analysis. **(C)** Network topology metrics for tissue-associated microbiota. **(D)** Network robustness assessment by simulated node removal using natural-connectivity analysis. In panel C, boxes indicate the interquartile range (25th–75th percentiles), and horizontal lines within boxes indicate the median. Whiskers indicate the most extreme observations within 1.5 times the interquartile range below the first quartile and above the third quartile. Statistical significance is indicated as follows: ns, not significant; *p <0.05; **p <0.01. NPC, nasopharyngeal carcinoma; NLH, nasopharyngeal lymphoid hyperplasia.

### NPC tissue microbial networks show greater apparent complexity and lower inferred robustness

To explore microbial association patterns, we constructed co-occurrence networks using the ggClusterNet R package (Wen et al., 2022). Swab-derived networks contained more nodes and edges than tissue-derived networks, and NPC-derived networks contained more nodes and edges than NLH-derived networks (**Figure 3B**; **Supplementary Table S4**). The NPC tissue network contained more clusters than the NLH tissue network (p <0.01; **Figure 3C**), suggesting a more heterogeneous set of inferred microbial associations. However, the NPC tissue network showed lower edge density (p <0.01) and longer average path length (p <0.01) than the NLH tissue network (**Figure 3C**). Natural-connectivity analysis under simulated node removal further suggested lower inferred robustness in the NPC tissue network (**Figure 3D**). Because these networks were inferred from compositional 16S profiles, the results should be viewed as hypothesis-generating evidence of altered microbial association structure rather than direct proof of ecological instability or interspecies interaction.

### Tissue-associated bacterial signatures show stronger apparent classification performance than biopsy-surface signatures

Species-level taxonomic assignments that differed between NPC and NLH in swab and tissue samples were screened with LEfSe. This analysis identified 11 species-level assignments enriched in SWAB_NLH, 8 in SWAB_NPC, 1 in TISSUE_NLH, and 12 in TISSUE_NPC (nominal p <0.05, LDA score >3; **Figure 4A**). Exploratory random-forest classifiers were then fitted separately using swab- derived and tissue-derived disease-associated features. ROC analysis showed stronger apparent discrimination for the tissue-based model (AUC = 0.927) than for the swab-based model (AUC = 0.408; **Figure 4B**). Sample-wise heat maps were consistent with these results: swab- derived disease-associated features showed substantial interindividual heterogeneity and weak group separation (**Figure 4C**), whereas tissue-derived disease-associated features showed more coherent NPC-associated shifts and higher model-importance scores (**Figure 4D**). These findings suggest that tissue-associated bacterial features captured stronger within-cohort separation between NPC and NLH than biopsy-surface features.

**Figure 4:**
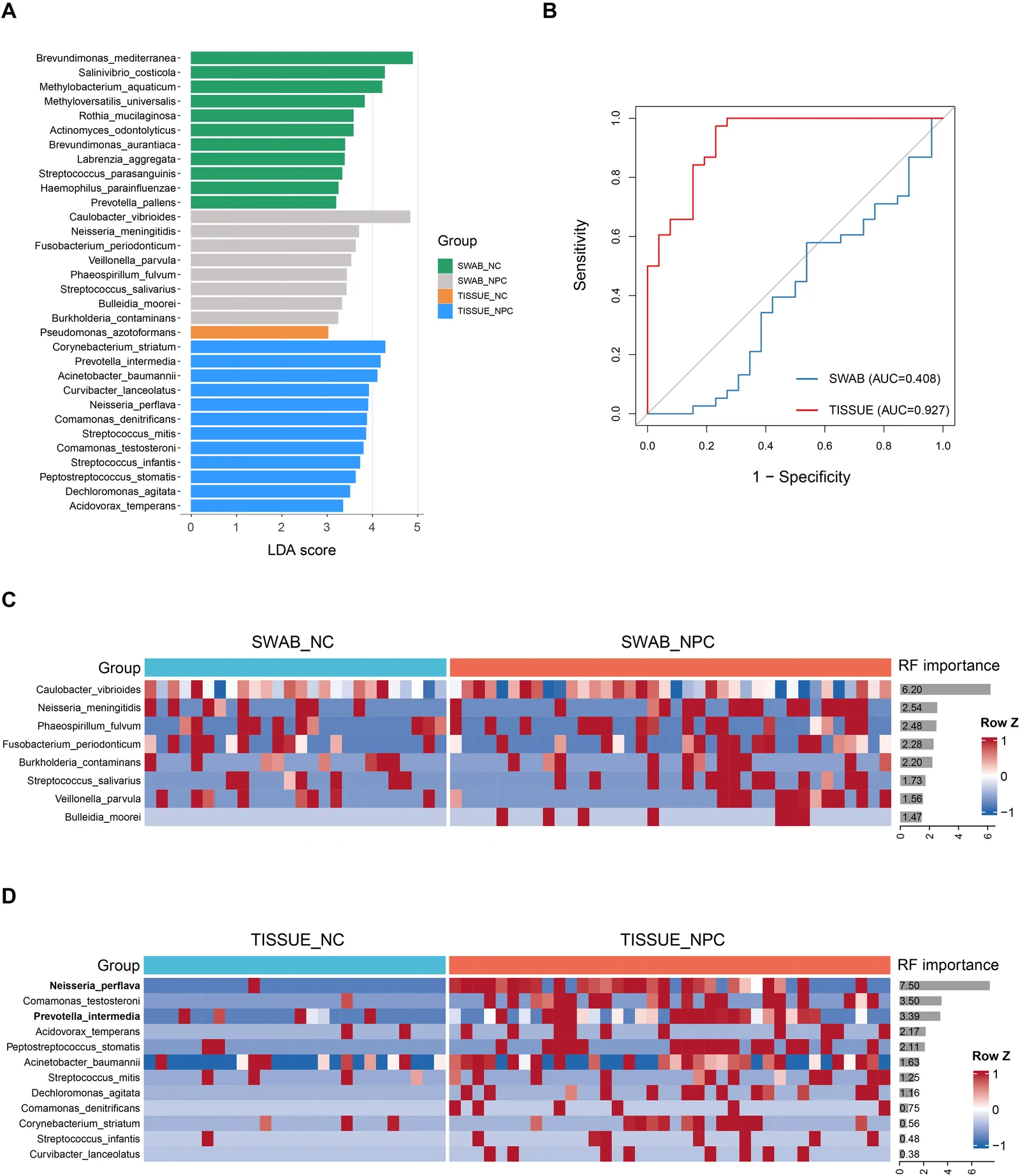
Tissue-derived candidate microbial features show stronger internal NPC/NLH separation than biopsy-surface-derived features. **(A)** LEfSe identified differentially enriched species-level assignments across groups (criteria: nominal p <0.05, LDA >3): 11 in SWAB_NLH, 8 in SWAB_NPC, 1 in TISSUE_NLH, and 12 in TISSUE_NPC. **(B)** Exploratory random-forest classifiers fitted with LEfSe-selected taxa showed stronger apparent performance for tissue-derived features (AUC = 0.927) than for swab-derived features (AUC = 0.408). Sample-wise heatmaps (row z-scores) of LEfSe-selected taxa in swabs **(C)** and tissues **(D)**. Side bars indicate random-forest feature importance. Tissue-derived disease-associated features display more coherent NPC-associated shifts, consistent with the ROC results. LEfSe, linear discriminant analysis effect size; LDA, linear discriminant analysis; RF, random forest; AUC, area under the ROC curve; NC denotes NLH control; NPC, nasopharyngeal carcinoma; NLH, nasopharyngeal lymphoid hyperplasia.

### Intratumoral bacteria associated with EBV state, oncogenic pathway, and immune-cell infiltration

Bulk RNA-seq was generated from NPC and NLH tissue samples that were also profiled by 16S rRNA sequencing to quantify host and EBV transcripts. Consistent with established NPC biology, EBV transcript levels were higher in NPC than in NLH (**Supplementary Figure S2**). Gene set variation analysis (GSVA) of MSigDB Hallmark gene sets showed increased activity of proliferation and cell-cycle programs, including E2F, MYC, G2M checkpoint, and mTORC1 signaling, in NPC tissues. Additional upregulated programs included unfolded protein response, DNA repair, and glycolysis, whereas selected oxidative stress, peroxisomal metabolism, developmental, and humoral immune programs were lower in NPC (**Supplementary Figure S3**). CIBERSORTx deconvolution suggested a remodeled immune composition, including increased macrophage, proliferative B-cell, plasma-cell, and dysfunctional CD8 T-cell signatures, together with reduced total B-cell, FCRL4 and germinal-center B-cell, conventional CD4 T-cell, regulatory T-cell, CD8 T-cell, and plasmacytoid dendritic-cell signatures (**Supplementary Figure S4**).

We then restricted the correlation analyses to the NPC group, relating species-level bacterial abundance in tissue and paired biopsy-surface samples to EBV transcript levels, Hallmark pathway GSVA scores, and inferred immune-cell fractions from the corresponding NPC tissues (**Figure 5A**). The tissue-focused correlation map suggested that selected bacterial taxa co-occurred with EBV transcriptional activity, proliferation/stress-response pathways, and immune-cell remodeling. The tissue correlation network contained more significant cross-domain associations than the biopsy-surface network, leading us to focus on tissue-associated profiles (**Figure 5B**). Several NPC-enriched species-level assignments, including Comamonas spp., Streptococcus spp., Prevotella intermedia, Corynebacterium striatum, and Peptostreptococcus stomatis, showed associations with EBV transcripts and proliferation, cell-cycle pathway scores, inferred CD8 T-cell and plasmacytoid dendritic-cell signatures. Neisseria perflava showed extensive cross-domain connectivity, including associations with MYC target, G2M checkpoint, DNA-repair programs, and EBV transcripts such as A73 and RPMS1, and an association with inferred CD8 T-cell abundance (**Figures 5A, B**). Rather than indicating direct causality, these associations suggest that tissue-associated bacteria may mark an EBV-active and immune-remodeled tumor niche. Because the current data are based on 16S profiling and bulk RNA-seq, the directionality of these relationships cannot be resolved.

**Figure 5:**
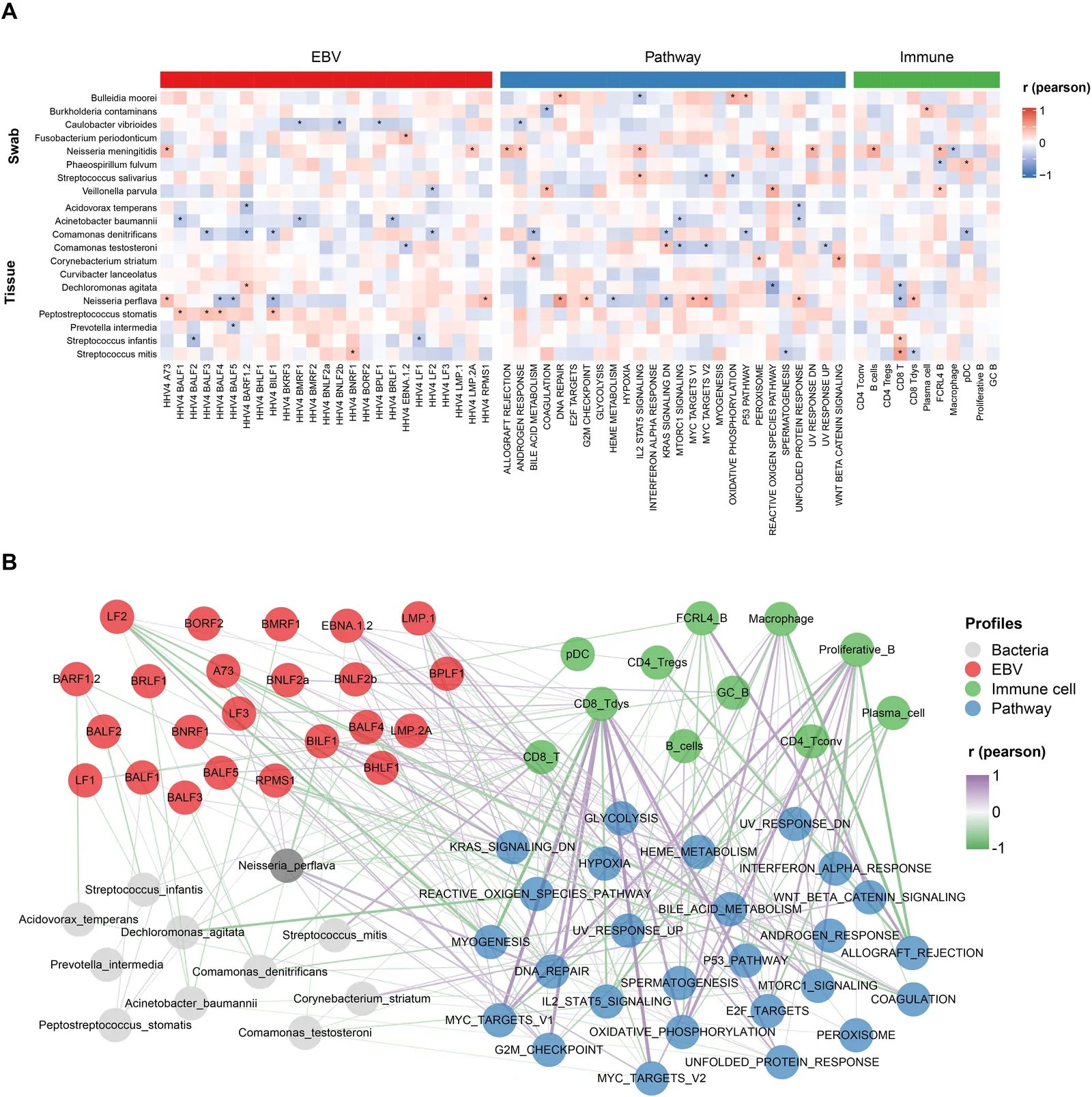
Exploratory cross-domain correlations among bacterial profiles, EBV transcripts, host pathway activity, and inferred immune-cell composition within the NPC group. **(A)** Pearson correlation heatmaps relating species-level bacterial abundance (rows) in NPC tissues and paired biopsy-surface swabs to EBV transcript levels (red bar), Hallmark pathway GSVA scores(blue), and CIBERSORTx-inferred immune-cell fractions (green) derived. Color denotes r (red, positive; blue, negative); asterisks indicate nominally significant correlations (p <0.05). **(B)** Tissue-focused correlation network integrating tissue-associated bacterial profiles (gray), EBV transcripts (red), Hallmark pathway scores(blue), and inferred immune-cell fractions (green) within the NPC group. Edges represent nominally significant Pearson correlations; edge color encodes sign and magnitude of (r) Selected NPC-enriched taxa, including Comamonas spp., Streptococcus spp., Prevotella intermedia, Corynebacterium striatum, and Peptostreptococcus stomatis, were associated with EBV and proliferative or cell-cycle programs and inversely associated with CD8 T-cell or pDC signatures. Neisseria perflava showed broad cross-domain connectivity. All analyses in panels **(A, B)** were restricted to patients with NPC; NLH samples were not included. NPC, nasopharyngeal carcinoma; NLH, nasopharyngeal lymphoid hyperplasia; EBV, Epstein-Barr virus.

Together, these analyses show that selected tissue-associated bacterial assignments covary with EBV transcriptional activity, host proliferative programs, and inferred immune composition in this cohort, but these relationships require targeted experimental validation.

## Discussion

Bacteria detected within tumor tissues can contribute to cancer biology in selected contexts, but interpretation of low-biomass tumor microbiome data requires careful control of sampling and contamination artifacts (Nejman et al., 2020; Fu et al., 2022). NPC develops at a mucosal site that is continuously exposed to environmental and commensal microbes, making it difficult to distinguish tissue-associated bacterial signals from biopsy-surface organisms. In this study, FISH, qPCR, and culture supported the presence of bacterial signal and viable bacteria in NPC tissue. Growth after ampicillin plus gentamicin exposure and suppression by doxycycline are compatible with a protected bacterial fraction. However, the culture assay was performed only under aerobic conditions and recovered colonies were not taxonomically identified. Therefore, culture positivity should be interpreted as orthogonal evidence of bacterial viability, but not as independent validation of the specific taxa detected by 16S rRNA sequencing.

Our study should also be distinguished from the recent work by Liao et al., which focused on oral-to-nasopharyngeal microbial translocation in NPC using paired oral and nasopharyngeal samples, source-tracking, culturomics, and strain-level comparisons (Liao et al., 2024). In contrast, the present study did not aim to infer oral microbial origin or microbial migration. Instead, we examined matched biopsy-surface swabs and underlying tissue from the same excised nasopharyngeal lesion to determine whether the surface compartment represents the tissue-associated microbial signal. Therefore, the main contribution of our study is the demonstration of lesion-level compartmentalization of recoverable microbial profiles and their exploratory cross-domain associations with EBV and host transcriptional states. Previous studies using superficial oral, nasal, or nasopharyngeal mucosal swabs have reported differences between NPC and non-malignant controls and have proposed microbiome-based screening strategies (Hao et al., 2022).Our biopsy-surface swabs did not show strong separation between NPC and NLH. This discrepancy is likely related to sampling design. In the present study, swabs were obtained from the surface of freshly excised, saline-rinsed biopsy specimens rather than from the nasopharyngeal mucosa *in vivo*. These samples therefore represent biopsy-surface communities and should not be equated with standard clinical nasopharyngeal swabs. This distinction limits conclusions about non-invasive screening but strengthens the within-specimen comparison between surface-associated and tissue-associated profiles.

NPC tissue samples showed higher alpha diversity than NLH tissue samples in this cohort. This may reflect features of the tumor microenvironment, including altered local immunity, tissue disruption, hypoxia, or nutrient availability; however, alternative explanations such as differences in tissue composition, bacterial load, and residual contamination cannot be excluded. Diversity patterns in cancer microbiome studies are inconsistent across tumor types, and methodological factors such as 16S target region, low-biomass decontamination, tissue processing, and taxonomic assignment can strongly influence apparent richness (Ott and Schreiber, 2006; Thyagarajan et al., 2020; Walker et al., 2020; Díaz et al., 2021; Esposito et al., 2022; Zhong et al., 2022; Rad et al., 2025).The diversity results should therefore be interpreted as a disease-associated pattern within this dataset rather than as direct evidence that microbial diversity contributes to NPC pathogenesis. Detailed lifestyle and exposure variables, including smoking, alcohol consumption, diet, oral-health status, and recent antibiotic use, were not available; residual confounding by these factors therefore cannot be excluded.

The co-occurrence analysis suggested that NPC tissue profiles contained a more clustered but less densely connected network than NLH tissue profiles. Such patterns may indicate altered microbial association structure in the tumor compartment. However, these networks were inferred from relative-abundance 16S data and cannot establish direct microbial interactions, ecological stability, or causal effects on tumor biology. The network results are best used to prioritize hypotheses about microbial community organization in NPC tissue.

The cross-domain analysis linked selected NPC-enriched bacterial assignments with EBV transcripts, proliferative host programs, and lower inferred cytotoxic or plasmacytoid dendritic-cell signatures. These findings are biologically plausible in light of prior evidence that microbes and oncogenic viruses can influence epithelial signaling and immune responses (Fuks et al., 2018; Cao et al., 2024; Liao et al., 2024). First, EBV-associated epithelial transformation may alter the local tissue environment through changes in nutrient availability, epithelial barrier function, inflammation, hypoxia, and immune surveillance, thereby creating conditions permissive to selected bacteria. Second, bacterial components may activate innate immune pathways, epithelial stress responses, or inflammatory signaling, which could in turn interact with viral and host transcriptional programs. Third, the observed bacterial signatures may reflect colonization of a pre-existing tumor niche rather than direct bacterial effects on tumor biology. As a mucosa-associated Gram-negative bacterium, N. perflava could plausibly interact with epithelial and immune pathways through microbial envelope components, local inflammatory signaling, or nitrogen/nitrosative metabolism. Nevertheless, the present data are correlative. Bulk RNA-seq deconvolution can be affected by tumor purity, stromal content, lymphoid tissue composition, and differences between NPC and NLH samples. The observed associations therefore do not demonstrate that bacteria and EBV act in concert or that bacteria directly suppress anti-tumor immunity. Targeted validation using bacterial qPCR/FISH, spatial co-localization, isolate-level characterization, and epithelial–EBV–bacteria co-culture models will be required to determine whether this taxon has a functional role in NPC or simply marks an EBV-conditioned tumor microenvironment.

Several limitations should be considered. The cohort was modest in size and drawn from a single setting, potentially limiting the detection of smaller effects. Nonsignificant findings should therefore not be interpreted as evidence of equivalence between groups. NLH is an inflammatory control rather than histologically normal nasopharyngeal mucosa. In this study, NLH was included as a histologically benign, nonmalignant inflammatory comparator rather than as a precursor lesion of NPC. Accordingly, the NPC–NLH comparison represents malignant versus nonmalignant inflammatory nasopharyngeal lesions, not a longitudinal disease-progression series or a comparison with healthy mucosa. Biopsy-surface swabs were collected from excised specimens and therefore do not represent conventional *in vivo* nasopharyngeal swabs. Despite the additional PBS rinses after surface sampling, a contribution from residual surface-adherent bacteria to the tissue-derived profiles cannot be excluded. The study used 16S rRNA profiling, so species-level assignments should be interpreted cautiously, particularly for closely related taxa. In addition, although bacterial culture supported the presence of viable culturable bacteria in NPC tissue or dissociated cell suspensions, the recovered colonies were not subjected to taxonomic identification. Thus, the culture results cannot validate the identities of specific sequencing-derived taxa. Future studies should combine aerobic and anaerobic culture with isolate-level identification by MALDI-TOF MS, 16S rRNA Sanger sequencing, or whole-genome sequencing. Although negative controls were included, low-biomass tissue microbiome studies remain vulnerable to reagent and environmental contamination. The random-forest classifier was exploratory and lacks independent validation. Correlation and network analyses were based on bulk tissue profiles and cannot establish causality. Larger cohorts, orthogonal taxonomic validation, spatial localization, and mechanistic models will be needed to test whether the candidate bacterial features identified here influence EBV-related signaling or immune states in NPC. Larger independent cohorts with balanced stage distributions, standardized clinical metadata, and external validation will be required before the clinical utility of either tissue-derived or biopsy-surface-derived microbial signatures can be claimed.

## Conclusions

By integrating 16S rRNA profiling with matched bulk RNA-seq, we demonstrated that nasopharyngeal carcinoma harbors a tissue-resident microbiome that is compositionally distinct from both the inflammatory control tissue (NLH) and surface swabs. In our cohort, tissue-derived microbial signatures effectively distinguished NPC from NLH. Furthermore, cross-domain analyses indicated coordinated variation among intratumoral bacteria, EBV transcripts, host proliferation programs, and immune composition, whereas the network topology indicated a complex yet fragile intratumoral ecology. These correlative findings suggest tissue-resident microbial features as candidate biomarkers and provide a strong impetus for mechanistic studies of microbe–EBV–host interactions in nasopharyngeal carcinoma.

## Data Availability

The raw data were deposited in the Genome Sequence Archive (Genomics, Proteomics & Bioinformatics 2025) in National Genomics Data Center (Nucleic Acids Res 2025), China National Center for Bioinformation / Beijing Institute of Genomics, Chinese Academy of Sciences that are publicly accessible at https://ngdc.cncb.ac.cn/gsa-human or https://ngdc.cncb.ac.cn/gsa. The BioProject accession is PRJCA042059, including RNA-Seq data (HRA014152) and 16S rRNA Seq data (CRA032474).
