# Supplementary material for: Paired biopsy-surface and tissue microbiome profiling reveals tissue-associated bacterial signatures in nasopharyngeal carcinoma
Source: Front Cell Infect Microbiol. 2026 Oct 14;16:1892233. doi: 10.3389/fcimb.2026.1892233 (PMC13713345; doi:10.3389/fcimb.2026.1892233)
Supplement: Supplementary file 1 [file DataSheet1.pdf]

Supplementary Material

Supplementary Table 1: Sequencing quality control data of paired nasopharyngeal tissue biopsies and matching surface swabs collected from NPC patients (n = 38) and NLH controls (n = 26) for 16S rRNA sequencing.

Supplementary Table 2: The baseline characteristics of the 38 patients with NPC and 26 participants with benign nasopharyngeal lymphoid hyperplasia (NLH).

Supplementary Table 3: Alpha diversity of the nasopharyngeal microbiome in NPC vs. NLH tissues and swabs.

Supplementary Table 4: Microbial co-occurrence network metrics for NPC and NLH groups. Key network properties (number of nodes, edges, clusters, edge density, average path length, and natural connectivity) are listed for co-occurrence networks constructed from NPC tumor microbiota and NLH tissue microbiota.

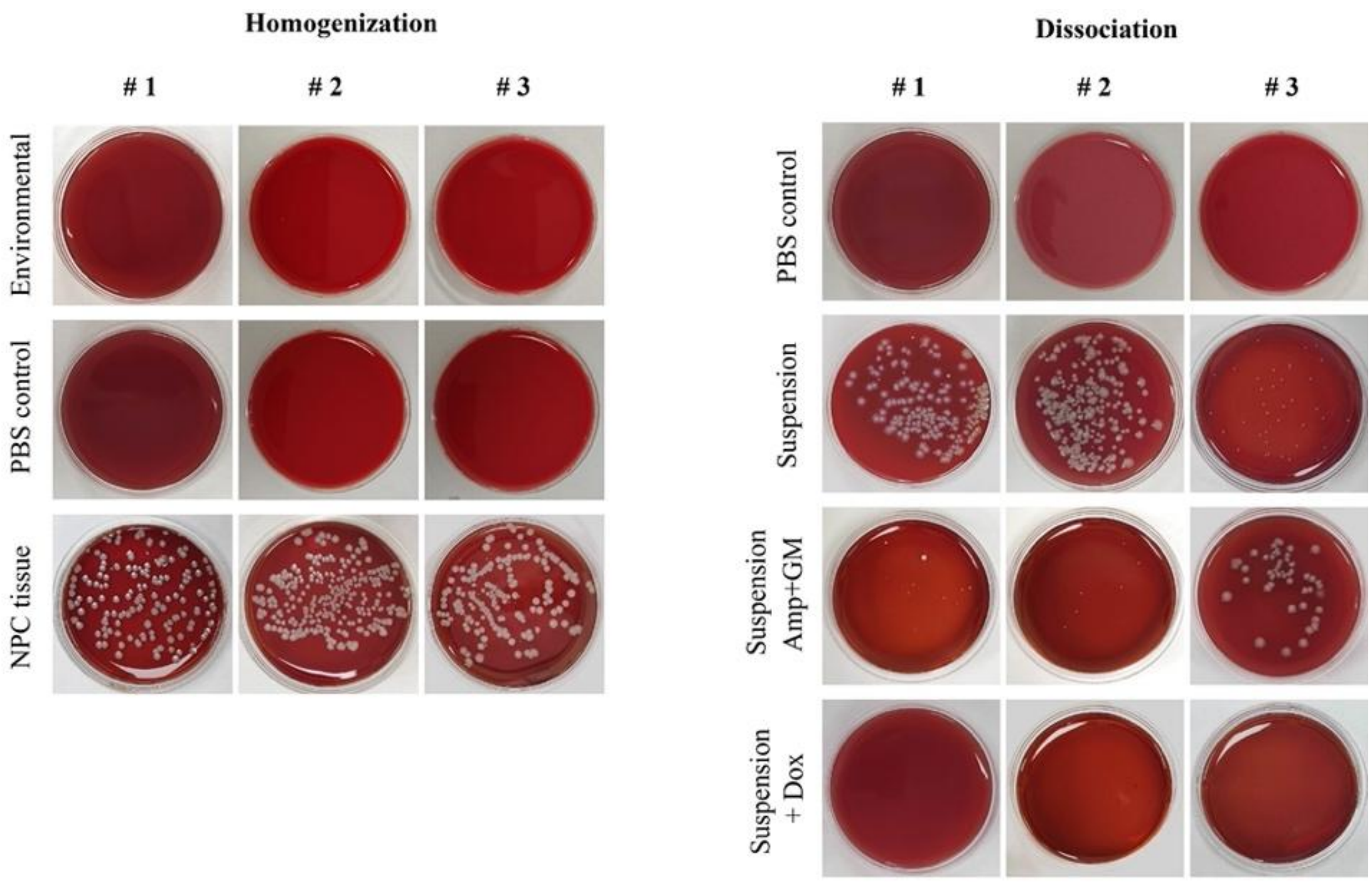

Supplementary Figure S1. Culture-based validation of viable, partially-intracellular bacteria in nasopharyngeal carcinoma (NPC)

Representative Columbia blood agar cultures (5% sheep blood; aerobic, 37 °C, 5% CO_2) from three independent experiments (#1–#3). Left (homogenization): NPC tissue homogenates yielded abundant colonies, whereas the environmental and PBS controls were sterile. Right (dissociation): Single-cell suspensions produce colonies without antibiotics and after treatment with cell-impermeable ampicillin + gentamicin (Amp+GM), but not after treatment with cell-penetrating (Dox). The persistence of growth with Amp+GM and its ablation by Dox indicated that a fraction of tumor-resident bacteria were intracellular.

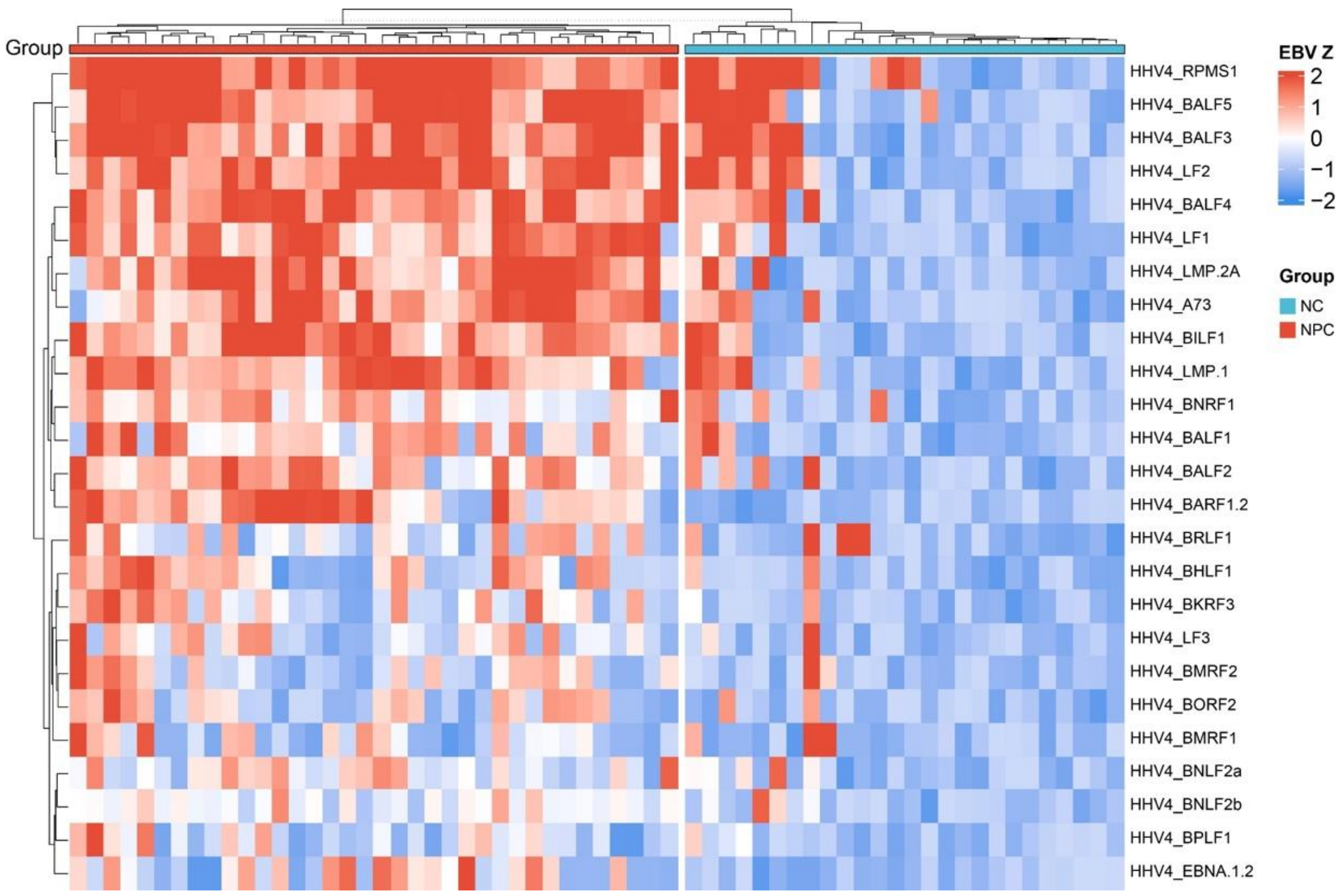

Supplementary Figure S2. EBV transcriptional activity in NPC versus NLH Heatmap of EBV (*HHV-4*) gene expression across individual tissue samples from the same cohort used for 16S profiling. Values are gene-wise *z*-scores (red, higher; blue, lower). Columns (samples) and rows (genes) are hierarchically clustered, with the top bar indicating the disease group (NPC vs. NLH). NPC tissues show globally-elevated EBV transcripts relative to NLH. NPC, nasopharyngeal carcinoma; NLH, nasopharyngeal lymphoid hyperplasia; EBV, Epstein–Barr virus

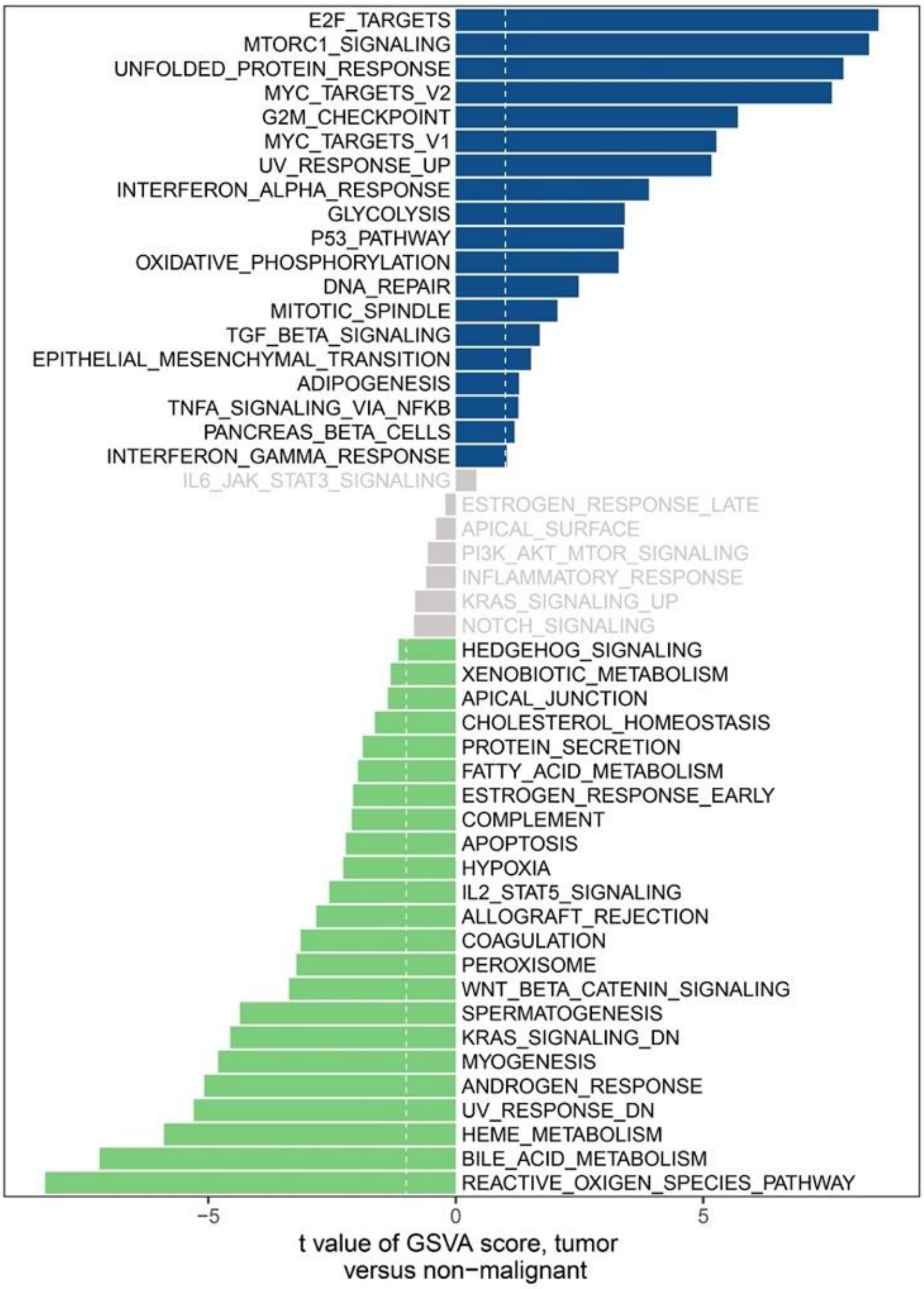

Supplementary Figure S3. Hallmark pathway activity (GSVA) in NPC versus NLHGSVA scores for MSigDB Hallmark gene sets were computed from bulk RNA-sequencing. Bars display the *t*-statistic for NPC minus NLH (positive/blue = higher in NPC; negative/green = lower in NPC). Proliferation and cell-cycle programs (e.g., E2F, MYC, G2M, MTORC1) and stress/metabolic pathways (e.g., unfolded protein response, DNA repair, glycolysis, oxidative phosphorylation) are increased in NPC, whereas oxidative-stress/peroxisomal and several metabolic/immune programs are reduced. GSVA, gene set variation analysis; MSigDB,

molecular signature database; NPC, nasopharyngeal carcinoma; NLH, nasopharyngeal lymphoid hyperplasia

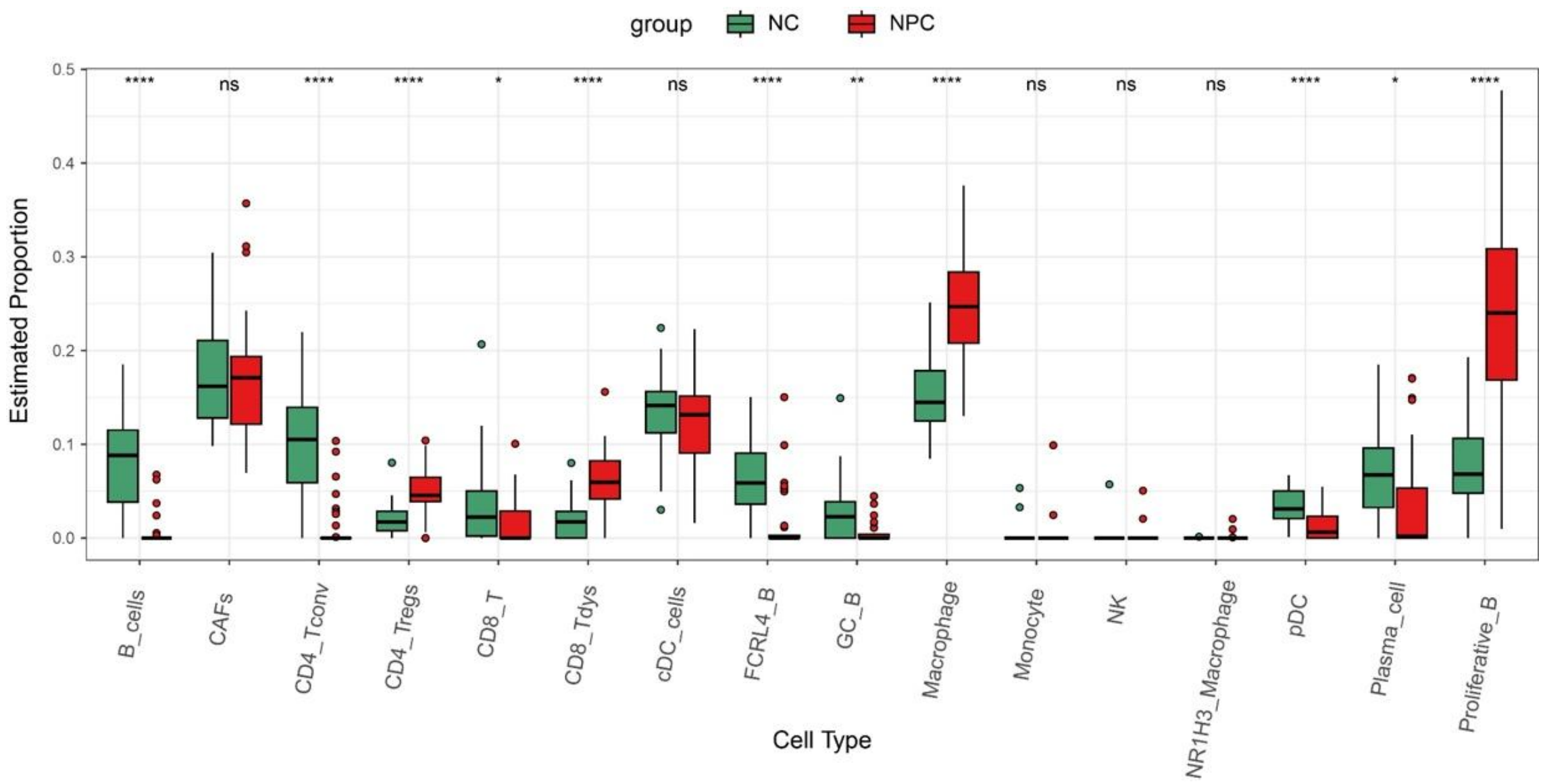

Supplementary Figure S4. Immune-cell landscape inferred from bulk RNA-sequencing

CIBERSORTx deconvolution estimates the relative abundance of immune and stromal populations in NPC and NLH tissues. Boxplots show per-sample proportions; asterisks denote between-group significance and "ns" indicates non-significant (statistical details in Methods). NPC is characterized by increased macrophages, proliferative B cells and plasma cells, with reduced total B cells (including FCRL4^{+} and germinal-center subsets), conventional CD4^{+} T cells, regulatory T cells, CD8^{+} T cells, and plasmacytoid dendritic cells. Abbreviations: CIBERSORTx, cell-type identification by estimating relative subsets of RNA transcripts; pDC, plasmacytoid dendritic cell; NPC, nasopharyngeal carcinoma; NLH, nasopharyngeal lymphoid hyperplasia; CAFs, cancer-associated fibroblasts
